# The variability of *SMCHD1* gene in FSHD patients: evidence of new mutations

**DOI:** 10.1093/hmg/ddz239

**Published:** 2019-10-10

**Authors:** Claudia Strafella, Valerio Caputo, Rosaria Maria Galota, Giulia Campoli, Cristina Bax, Luca Colantoni, Giulietta Minozzi, Chiara Orsini, Luisa Politano, Giorgio Tasca, Giuseppe Novelli, Enzo Ricci, Emiliano Giardina, Raffaella Cascella

**Affiliations:** 1 Genomic Medicine Laboratory UILDM, Santa Lucia Foundation, Rome, 00142, Italy; 2 Department of Biomedicine and Prevention, Tor Vergata University, Rome, 00133, Italy; 3 Department of Veterinary Medicine (DIMEVET), University of Milan, Milan, 20100, Italy; 4 vCardiomyology and Medical Genetics, Department of Experimental Medicine, University of Campania *Luigi Vanvitelli*, Naples, 80131, Italy; 5 Unità Operativa Complessa di Neurologia, Fondazione Policlinico Universitario A. Gemelli IRCCS, Rome, 00168, Italy; 6 Neuromed Institute IRCCS, Pozzilli, 86077, Italy; 7 Istituto di Neurologia, Università Cattolica del Sacro Cuore, Rome, 00168, Italy; 8 Department of Biomedical Sciences, Catholic University Our Lady of Good Counsel, Tirana, 1000, Albania

## Abstract

In this study, we investigated the sequence of (*Structural Maintenance of Chromosomes flexible Hinge Domain containing* 1) *SMCHD1* gene in a cohort of clinically defined FSHD (facioscapulohumeral muscular dystrophy) patients in order to assess the distribution of *SMCHD1* variants, considering the *D4Z4* fragment size in terms of repeated units (RUs; short fragment: 1–7 RU, borderline: 8-10RU and normal fragment: >11RU). The analysis of *SMCHD1* revealed the presence of 82 variants scattered throughout the introns, exons and 3’untranslated region (3′UTR) of the gene. Among them, 64 were classified as benign polymorphisms and 6 as VUS (variants of uncertain significance). Interestingly, seven pathogenic/likely pathogenic variants were identified in patients carrying a borderline or normal *D4Z4* fragment size, namely c.182_183dupGT (p.Q62Vfs^*^48), c.2129dupC (p.A711Cfs^*^11), c.3469G>T (p.G1157^*^), c.5150_5151delAA (p.K1717Rfs^*^16) and c.1131+2_1131+5delTAAG, c.3010A>T (p.K1004^*^), c.853G>C (p.G285R). All of them were predicted to disrupt the structure and conformation of SMCHD1, resulting in the loss of *GHKL-ATPase* and *SMC hinge* essential domains. These results are consistent with the FSHD symptomatology and the Clinical Severity Score (CSS) of patients. In addition, five variants (c.^*^1376A>C, rs7238459; c.^*^1579G>A, rs559994; c.^*^1397A>G, rs150573037; c.^*^1631C>T, rs193227855; c.^*^1889G>C, rs149259359) were identified in the 3′UTR region of *SMCHD1*, suggesting a possible miRNA-dependent regulatory effect on FSHD-related pathways. The present study highlights the clinical utility of next-generation sequencing (NGS) platforms for the molecular diagnosis of FSHD and the importance of integrating molecular findings and clinical data in order to improve the accuracy of genotype–phenotype correlations.

## Introduction

The application of molecular genetics into the clinical practice highlighted the existence of a large gap between the genotype and phenotype in many human disorders [1–2]. This is particularly true for neuromuscular disorders, which include a heterogeneous group of pathologies characterized by progressive weakness and wasting of proximal and/or distal muscles [3–4]. The phenotypic overlapping symptoms, the limited availability of muscle biopsies and the multisystemic events occurring in neuromuscular disorders raised the need for a multidisciplinary approach to provide an accurate diagnosis and allow targeted interventions according to the profile of each patient [3–4]. Our group is one of the two Italian Reference Centers for the genetic characterization of facioscapulohumeral muscular dystrophy (FSHD, OMIM #158900). FSHD affects approximately 1 in 8300 individuals [5–7]. The disease is characterized by clinical variability and incomplete penetrance, which can lead to asymptomatic or wheelchair-dependent individuals [2]. In the initial stage of FSHD, patients experience a progressive weakness of scapular girdle, facial and humeral muscles. Later, the weakness can extend to the muscles of trunk and of lower extremities, leading thereby to loss of ambulation in 20% of cases [8–9]. Two forms of FSHD are known, namely FSHD1 and FSHD2, which are characterized by identical clinical features but different genetic signatures [2].

FSHD1 accounts for approximately 95% of cases, and it is associated with a contraction of a microsatellite repeat array on the 4q35 chromosome [2]. This region is 3.3 kb long and is referred to as *D4Z4* region. In healthy individuals, the repeat consists of 11 to 100 repeated units (RUs), whereas it is 1–10 RU in FSHD1 patients. The array contraction results in the hypomethylation of *D4Z4* and, consequently, in the abnormal expression of *Double Homeobox Protein 4* (*DUX4*) that is toxic for muscle cells [10–11]. However, it is important to remark that as many as 2% of the general population presents 8–10 RU without being affected [2]. These findings suggested that the etiopathogenesis of FSHD might not be based on the *D4Z4* contraction on 4q35 alone, but to a combination of specific genetic and epigenetic signatures, which create a permissive background for the development of disease.

Approximately 5% of patients show clinical symptoms typical of FSHD, without carrying a short allele on the *D4Z4* repeat array. This form is clinically identical to FSHD1 but genetically distinct, and it is termed FSHD2 (OMIM #158901) [12]. FSHD2 has been associated with mutations in the *Structural Maintenance of Chromosomes flexible Hinge Domain containing 1* (*SMCHD1*, 18p11.32, OMIM #614982) gene. *SMCHD1* consists of 48 exons and encodes the homonymous protein belonging to the highly conserved SMC protein family, although it is also regarded as a member of the human microrchidia (MORC) family. Both groups of proteins are involved in the epigenetic regulation of the chromatin status [13–14]. In fact, *SMCHD1* is mainly involved in the regulation of high-order chromosome structures, in the inactivation of X chromosome and in the epigenetic regulation of chromatin repression [14–15]. In particular, *SMCHD1* contributes to the somatic repression of *DUX4* by directly binding to the *D4Z4* repeat array [16]. SMCHD1 protein contains an N-terminal *GHKL-ATPase* domain and a non-canonical C-terminal *SMC hinge* domain, both flanked by coiled-coil regions and uncharacterized domains. These functional domains are involved in the homodimerization of the protein, which is regarded as a fundamental mechanism for its activity [15]. The mutational spectrum of *SMCHD1* includes small deletions, splice site mutations and missense mutations [17]. These mutations decrease the binding activity of SMCHD1, resulting in *D4Z4* hypomethylation and incomplete repression of *DUX4*, which is thereby expressed in muscle tissue [16]. In addition, mutations of *SMCHD1* have also been shown to act as disease modifiers in FSHD patients carrying short or borderline *D4Z4* fragments [2,18]. However, the lack of a precise genotype–phenotype correlation in many cases requires a more comprehensive genetic analysis of both *D4Z4* alleles and *SMCHD1*. In this work, we report the sequence analysis of *SMCHD1* in a cohort of clinically defined FSHD patients in order to assess the distribution of *SMCHD1* variants, considering the *D4Z4* size (short fragment: 1-7RU, borderline fragment: 8-10RU and normal fragment: >11RU).

## Results

NGS and traditional methodologies proved to be useful to characterize *D4Z4* fragment, 4qA and *SMCHD1* sequence in the patient’s cohort. We selected a cohort of patients representative of the three categories of patients in terms of fragment size: a number of 23 patients presenting a normal range (>11RU), 13 subjects with borderline (8-10RU) and 33 patients with short fragment (1-7RU). All the patients resulted to be 4qA-positive. Successively, the sequence analysis of *SMCHD1* was performed in all patients. The extensive analysis of the *SMCHD1* sequence revealed the presence of 82 variants scattered throughout the introns, exons and 3′UTR regions of the gene. The assessment of frequency distribution and the analysis of bioinformatics data allowed describing eight exonic variants as benign or likely benign (Supplementary Material, Table 1), whereas six exonic variants were classified as variants of uncertain significance (VUS, Supplementary Material, Table 1) and need to be further investigated. In addition, 56 intronic variants were detected, although none of them has been shown to affect the splicing activity (Supplementary Material, Table 2). The frequency distributions of the previously described variants are consistent with the frequency distributions observed in the general population and are not correlated with any fragment size category. Moreover, the analysis of the *SMCHD1* sequence pointed out the attention on seven pathogenic and likely pathogenic variants in seven FSHD patients carrying a borderline or normal sized *D4Z4* fragment, which are c.182_183dupGT (p.Q62Vfs^*^48), c.2129dupC (p.A711Cfs^*^11), c.3469G>T (p.G1157^*^), c.5150_5151delAA (p.K1717Rfs^*^16) and c.1131+2_1131+5delTAAG, c.3010A>T (p.K1004^*^), c.853G>C (p.G285R) (Table 1). All of the variants were confirmed by direct sequencing. In these cases, the molecular analysis was consistent with the clinical diagnosis of FSHD and the Clinical Severity Score (CSS, Table 1). The pattern of muscle involvement found on magnetic resonance imaging (MRI) was consistent with that described in FSHD [19–20]. In addition, the analysis performed on muscle biopsies allowed excluding pathological abnormalities and protein deficiencies associated with other muscular dystrophies.

**Table 1:** List of *SMCHD1* (NM_015295.2) pathogenic and likely pathogenic mutations identified in seven FSHD patients, considering their age, Clinical Severity Score (CSS) and *D4Z4* fragment size

**Patient**	**Age (years)**	**CSS**	***D4Z4* size**	**4qA**	***SMCHD1*_variant position**	***SMCHD1*_HGVS nomenclature**
I	28	1.5	10RU	+	18:2656257_2656258	c.182_183dupGT
II	73	3	9RU	+	18:2707627_2707628	c.2129dupC
III	52	4	8RU	+	18:2739473	c.3469G>T
IV	62	4	>11RU	+	18:2697122_2697125	c.1131+2_1131+5delTAAG
V	24	−	8RU	+	18:2772345_2772346	c.5150_5151delAA
VI	75	3	>11RU	+	18:2729369	c.3010A>T
VII	39	3	9RU	+	18:2688725	c.853G>C

The bioinformatic characterization and the American College of Medical Genetics (ACMG) classification of the seven identified variants are reported in the following paragraphs. In addition, the last subsection has been focused on the bioinformatic analysis of the variants detected in the 3′UTR of *SMCHD1*.

### *SMCHD1*:c.182_183dupGT (p.Q62Vfs^*^48)

**Figure 1:**
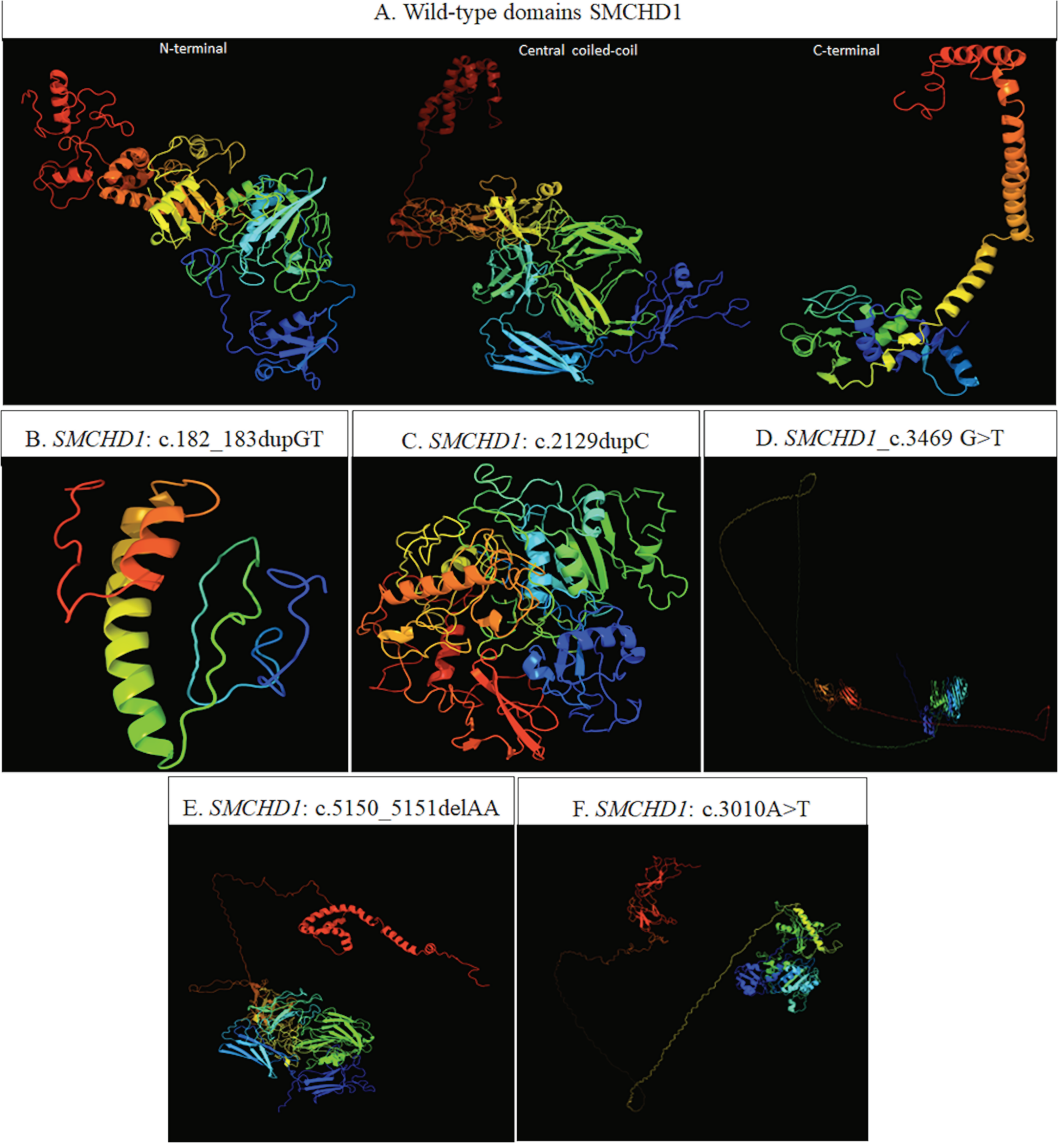
(**A**) Predicted conformation of the three wild-type domains of SMCHD1, based on the domain organization released by UniProt (entry: A6NHR9). In particular, the N-terminal region (1–702 AA) harboring the *GHKL-ATPase* domain (111–702 AA) is based on the template c5ix1A (PDB header: transcription; PDB molecule: MORC family CW-type zinc finger protein 3; PDBTitle: crystal structure of mouse Morc3 ATPase-CW cassette in complex with AMPPNP and H3K4me3 peptide). The central coiled-coil domain (703–1719 AA) is based on the template c4e9lA (PDB header: cell adhesion; PDB molecule: attaching and effacing protein, pathogenesis factor; PDBTitle: FdeC, a novel broadly conserved *Escherichia coli* adhesin eliciting protection against urinary tract infections). The C-terminal region (1720–2005 AA) harboring the *SMC hinge* domain (1720-1847AA) is based on c2wd5A (PDB header: cell cycle chain: A: PDB molecule: structural maintenance of chromosomes protein 1a; PDBTitle: SMC hinge heterodimer (mouse). (**B–F**) 3D model predicted by Phyre2 tool. The structure resulting from the presence of c.182_183dupGT (**B**) is based on the template d1e9ya1 (fold: beta-clip superfamily: urease, beta-subunit). The structures resulting from the presence of the c.2129dupC and c.3469G>T (**C** and **D**, respectively) are based on the template c5ix1A (PDB header: transcription. PDB molecule: MORC family CW-type zinc finger protein 3). The structure resulting from c.5150_5151delAA (**E**) is based on the template c4e9IA (PDB header: cell adhesion; PDB molecule: attaching and effacing protein, pathogenesis factor). The structure referred to the c.3010A>T (**F**) is based on the template c5ix1A (PDB header: transcription. PDB Molecule: MORC family CW-type zinc finger protein). The 3D model simulation of the *SMCHD1*_c.1131+2_1131+5delTAAG is not available because the amino acid sequence alteration following this variant cannot be predicted.

The insertion variant c.182_183dupGT is localized in the exon 1 of *SMCHD1* and has been found in one individual at the heterozygous state. This variant was not present in none of the annotation databases and has been predicted to be disease-causing by MutationTaster. In fact, the c.182_183dupGT may create a premature termination codon (PTC), causing the termination of the amino acid sequence at the 109th amino acid (instead of the canonical 2006th codon), leading thereby to the activation of nonsense-mediated mRNA decay (NMD) [11]. The analysis of this variant by the SMART prediction tool revealed that the truncated protein may result in the loss of its essential functional domains, namely the *GHKL-ATPase* and the *SMC hinge* domains. This alteration was also visible by comparing the wild-type (Fig. 1A) and variant 3D model simulations obtained by Phyre2 tool. Figure 1B illustrates the strong alteration of the protein secondary structures and of the subsequent conformation of the variant protein in contrast to the wild-type structure. The analysis on Human Splicing Finder (HSF) indicated that the variant may impact the splicing as well, causing the disruption of a donor splice site, the creation of an Exonic Silencer Site (ESS) or the activation of a cryptic exonic donor site. According to ACMG, the c.182_183dupGT has been classified as a likely pathogenic variant (Table 2). As a matter of fact, c.182_183dupGT is a null variant potentially causing loss of function (LOF) of *SMCHD1* (PVS1), and it is absent in ExAc, GnomAD and 1000 Genomes Browser (PM2).

### *SMCHD1*:c.2129dupC (p.A711Cfs^*^11)

The insertion variant c.2129dupC has been found in the exon 16 of *SMCHD1* in a single individual at the heterozygous state. This variant has not been annotated in any of the online databases. MutationTaster prediction described the c.2129dupC as a disease-causing variant, since it may create a frameshift and, consequently, a PTC at the 721th amino acid and NMD [11]. The analysis of the variant effect by the SMART tool suggested that the truncated protein may lose the C-terminal *SMC hinge* domain. Consistently with this data, Phyre2 showed that the 3D model predicted for the variant protein appeared to have a more compressed conformation (Fig. 1C) with respect to the wild-type structure (Fig. 1A). The HSF analysis did not reveal a potential alteration of splicing. According to ACMG, c.2129dupC can be classified as a pathogenic variant (Table 2), since it is a null variant leading to a LOF of *SMCHD1* (PVS1); it is absent in ExAc, GnomAD and 1000 Genome Browser (PM2), and there is computational evidence supporting a deleterious effect on the gene product without benign-supporting predictions (PP3).

### *SMCHD1*:c.3469G>T (p.G1157^*^)

The variant c.3469G>T (p.G1157^*^) is situated within the exon 27 of *SMCHD1* and has been identified in a single patient at the heterozygous state. This variant has not been reported on the online annotation databases and has been predicted to have a damaging effect by MutationTaster. In fact, the c.3469G>T generates a frameshift, causing a PTC at the 1157th amino acid and probably triggering the NMD process [11]. In addition, the analysis of variant by SMART and Phyre2 showed the loss of the *SMC hinge* domain and, consequently, the disruption of a secondary structure and a partial relaxation of the tridimensional conformation of SMCHD1 (Fig. 1D). This variant has also been investigated by HSF, showing that it can affect splicing through the alteration of an exonic splicing enhancer (ESE) site. Following ACMG criteria, c.3469G>T can be described as a pathogenic variant (Table 2), since it is a null variant (PVS1), it is absent on ExAc, GnomAD and 1000 Genome Browser (PM2) and it has been predicted to be damaging for the gene or the gene product (PP3).

### *SMCHD1*:c.5150_5151delAA (p.K1717Rfs^*^16)

The c.5150_5151delAA has been detected in exon 41, in a single case at the heterozygous state. This variant was predicted to have a pathogenic effect, leading to NMD and causing the loss of the C-terminal *SMC hinge* domain [11]*.* Moreover*,* the 3D model obtained by Phyre2 highlighted the maintenance of the central coiled-coil domain conformation in the truncated protein (Fig. 1E). This variant has been described as a pathogenic variant (Table 2) in our previous work, in which we described an accurate genotype–phenotype correlation within the proband and his family [21]. However, we decided to include the sample even in the present study because we performed the 3D simulation of the variant protein and we evaluated the 3′UTR region of *SMCHD1*.

### *SMCHD1*:c.1131+2_1131+5delTAAG

The intronic c.1131+2_1131+5delTAAG variant has been found downstream exon 9 in one patient at the heterozygous state. This variant is novel and has been predicted to affect splicing and lead to NMD [11]. In addition, c.1131+2_1131+5delTAAG was not found in the annotation databases and has been reported as a disease-causing variant on MutationTaster. However, the prediction of the effect on the protein domains could not be performed because it is not possible to predict how the sequence and the reading frame may be altered following this variant, although it is likely to affect splicing. According to ACMG guidelines, the variant has been classified as likely pathogenic (Table 2), considering that it is a null variant (PVS1) and it has not been reported in ExAc, GnomAD and 1000 Genome Browser (PM2).

### *SMCHD1*:c.3010A>T (p.K1004^*^)

c.3010A>T has been localized in the exon 24 of *SMCHD1* in a single patient at the heterozygous state. This variant has not been reported on the online annotation databases and has been predicted to have a damaging effect by MutationTaster. In fact, the variant has been predicted to generate a PTC at the 1004th amino acid, probably triggering the NMD process [11]. The interrogation of HSF indicated that the variant may affect the splicing as well, causing the creation of an ESS or the alteration of the ESE site. In addition, the analysis performed by SMART and Phyre2 reported that the truncated protein may result in the loss of the *SMC hinge* domain and, consequently, in the disruption of the secondary structure and the tridimensional conformation of SMCHD1 (Fig. 1F). The ACMG classification of c.3010A>T described it as a pathogenic variant (Table 2), since it is a null variant (PVS1), it is absent on ExAc, GnomAD and 1000 Genome Browser (PM2) and it has been predicted to be damaging for the gene or the gene product (PP3).

**Table 2:** Prediction analysis and ACMG classification of the seven *SMCHD1* (NM_015295.2) mutations. ESS: exonic silencer site; ESE: exonic splicing enhancer; WT: wild-type

***SMCHD1* mutations**	**MutationTaster**	**SMART**	**Human splicing finder**	**ACMG**
c.182_183dupGT	Disease-causing	Loss of *GHKL-ATPase* domain and *SMC hinge* domain	Disruption of a donor splice site; activation of an exonic cryptic donor site or creation of an ESS	Likely pathogenic
c.2129dupC	Disease-causing	Loss of *SMC hinge* domain	No significant splicing motif alteration detected	Pathogenic
c.3469G>T	Disease-causing	Loss of *SMC hinge* domain	Alteration of an ESE	Pathogenic
c.5150_5151delAA	Disease-causing	Loss of *SMC hinge* domain	Creation of an ESS or alteration of an ESE	Pathogenic
c.1131+2_1131+5delTAAG	Disease-causing	NA	Alteration of the WT donor site	Likely pathogenic
c.853G>C	Disease-causing	No significant alteration of domain organization	No significant splicing motif alteration detected	Likely pathogenic
c.3010A>T	Disease-causing	Loss of *SMC hinge* domain	Creation of an ESS or alteration of an ESE	Pathogenic

### *SMCHD1*:c.853G>C (p.G285R)

**Figure 2:**
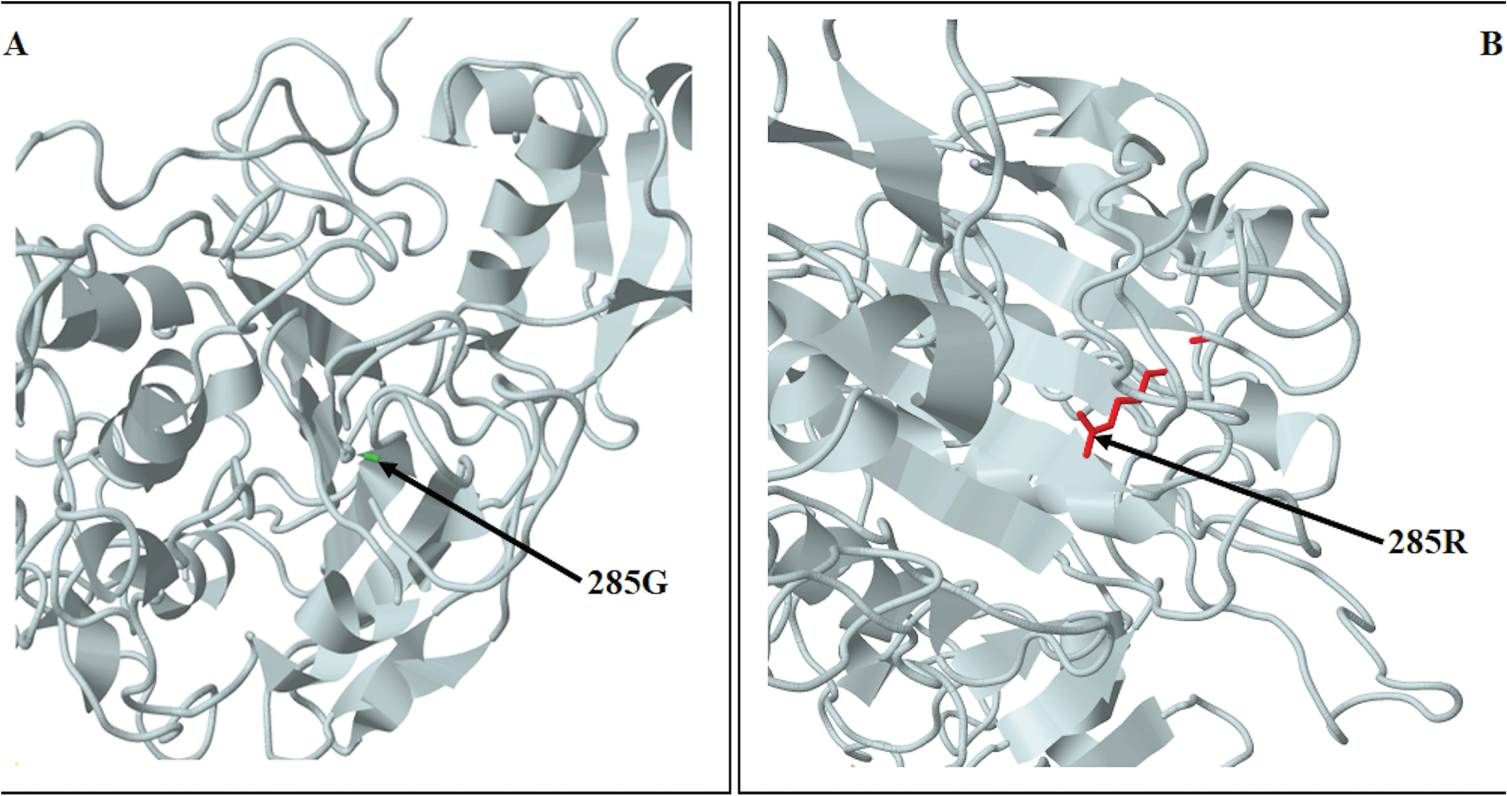
Predicted structure of the N-terminal region structure of SMCHD1 showing the amino acid change resulting from c.853G>C. The predicted models are based on the template c5ix1A (PDB header: transcription; PDB molecule: MORC family CW-type zinc finger protein 3; PDBTitle: crystal structure of mouse Morc3 ATPase-CW cassette in complex with AMPPNP and H3K4me3 peptide). (**A**) SMCHD1 structure showing the wild-type residue (G). (**B**) SMCHD1 structure showing the variant residue (R).

The variant c.853G>C has been detected in exon 7, in a single patient at the heterozygous state. It is a missense variant and has been described as disease-causing by MutationTaster. In fact, the variant produces an amino acid change in the *GHKL-ATPase* protein domain of SMCHD1. The HSF did not reveal a potential alteration of splicing. However, the predictive analysis performed on VarSite reported that the amino acid substitution may be highly negative in terms of conserved amino acid properties because of the change from a neutral (G) to a charged residue (R). Supporting this finding, interrogation of the Missense3D tool revealed a damaging effect on the protein structure resulting from the steric hindrance, the introduction of a buried charge and the substitution of a buried glycine residue, which, in turn, impairs the bending of the polypeptide chain (Fig. 2A and B). According to ACMG guidelines, c.853G>C could be likely pathogenic (Table 2), considering that it is located in a mutational hotspot within a functional domain of the protein (PM1); it is absent on ExAc, GnomAD and 1000 Genome Browser (PM2); it has been found in other affected family members (PP1) and has been predicted to be damaging for the gene or the gene product (PP3).

### Analysis of the 3′UTR of *SMCHD1*

The analysis of the 3′UTR of *SMCHD1* revealed different variants in our patient’s cohort. However, our attention was focused on c.^*^1376A>C (rs7238459), c.^*^1579G>A (rs559994), c.^*^1397A>G (rs150573037), c.^*^1631C>T (rs193227855) and c.^*^1889G>C (rs149259359), since the variant alleles may disrupt an existing binding site or create a novel binding site for different miRNAs (Table 3). The rs7238459 reported a MAF = 0.254 in our patient’s cohort, which overlaps the frequency observed in the general population (MAF = 0.257). According to PolymiRTS, the variant allele (C) of rs7238459 is able to disrupt a binding site for MIR7850 as well as to create a site for MIR6740.

**Table 3:** Bioinformatic prediction of 3′UTR variants altering the match to the miRNA seed region. MAF: minor allele frequency. ^*^Calculated on 69 patients, ^**#**^Referred to 1000 Genomes allele frequencies

**Genomic position**	**SNP**	**MAF FSHD** ^*^	**MAF EUR** ^**#**^	**Effect of the variant allele**	**Targeted miRNA**
18:2803926	rs7238459 (A/C)	C: 0.254	C: 0.257	Disruption of a conserved miRNA site	MIR-7850
				Creation of a new miRNA site	MIR-6740
18:2804129	rs559994 (G/A)	A: 0.268	A: 0.438	Creation of a new miRNA site	MIR-548AT
18:2804439	rs149259359 (G/C)	C: 0.007	C: 0.014	Disruption of a conserved miRNA site	MIR-3942
					MIR-4503
					MIR-4703
					MIR-6792
					MIR-95
				Creation of a new miRNA site	MIR-4477B
					MIR-651
					MIR-7856
18:2803947	rs150573037 (A/G)	G: 0.007	G: 0.000	Creation of a new miRNA site	MIR-515
					MIR-519D
					MIR-519E
					MIR-5695
18:2804181	rs193227855 (C/T)	T: 0.014	T:0.000	Disruption of a conserved miRNA site	MIR-548E
				Creation of a new miRNA site	MIR-495
					MIR-548AC
					MIR-548AE
					MIR-548AH
					MIR-548AJ
					MIR-548AM
					MIR-548AQ
					MIR-548D
					MIR-548H
					MIR-548 J
					MIR-548X
					MIR-548Z
					MIR-5688

The rs559994 had a MAF = 0.268 in our cases, which is lower in contrast to the frequency in the general population (MAF = 0.438). PolymiRTS interrogation revealed that the variant allele (A) may create a new binding site for MIR548AT.

rs150573037 has only been detected in a single patient (MAF = 0.007). Interestingly, frequency data for this single-nucleotide variation (SNV) are only available for the African population (MAF = 0.008) whereas it has not been observed in the European population to our knowledge. Prediction analysis indicated that the variant allele (G) of rs150573037 may generate new binding sites for MIR515, MIR519, MIR519E and MIR5695.

rs193227855 has been found in two patients (MAF = 0.014) in contrast to the lower frequency (MAF = 0.006) observed only in the control population of American Ancestry. Based on PolymiRTS prediction analysis, this variant may disrupt the binding site for MIR548E and create new binding sites for MIR495 and MIR548-family members.

Finally, rs149259359 has been reported in a single patient of the cohort and has been rarely observed in the general population (MAF = 0.014). Interestingly, this is the patient carrying the *SMCHD1*_c.5150_5151delAA variant and already described in our previous work [21]. The segregation analysis on the family members reported the heterozygous presence of rs149259359 in both the affected mother and maternal uncle. The analysis performed by PolymiRTS revealed that the variant allele (C) of rs149259359 may disrupt binding sites for MIR3942, MIR4503, MIR4703, MIR6792 and MIR95, whereas it may create novel binding sites for MIR4477B, MIR651 and MIR7856.

## Discussion

FSHD is one of the most difficult diseases to deal with, because of the complex genetic and epigenetic background underlying its etiopathogenesis. In fact, the variable penetrance and expressivity (observed either in related or unrelated patients) do not allow an accurate diagnosis, which is further complicated by the lack of a precise genotype–phenotype correlation [2,18]. We therefore decided to extend our study to the analysis of the *SMCHD1* sequence, which can be helpful for genotype–phenotype correlation in FSHD patients. On this subject, our previous work described the case of a patient presenting severe FSHD symptoms, in which preliminary genetic analysis did not clarify the phenotype [21]. In fact, a contracted *D4Z4* fragment was detected both in the affected proband and the healthy father, without explaining thereby the severe symptomatology of the proband and highlighting a reduced penetrance of disease within the family. The subsequent analysis of *SMCHD1* revealed the presence of a novel pathogenic variant in the proband, which was also detected in the mother and the maternal uncle who were both affected by mild FSHD symptoms without carrying a short *D4Z4* fragment. The severe phenotype of the proband may therefore be explained by the digenic inheritance of a contracted fragment and a *SMCHD1* variant [21]. In the present study, the analysis of the *SMCHD1* sequence reported 82 variants, which were localized throughout the introns, exons and 3′UTR regions of the genes. Among them, 64 were classified as polymorphisms with a frequency distribution overlapping those observed in the general population. These variants are probably not related with FSHD neither with the *D4Z4* fragment size, suggesting that they are not involved in disease etiopathogenesis. Moreover, five non-described VUS were also detected, but they need to be re-evaluated as more information and/or literature data will be collected concerning their potential clinical relevance in FSHD. In addition, none of them is correlated with a specific class of *D4Z4* fragment size.

Interestingly, seven pathogenic/likely pathogenic variants were identified by *SMCHD1* sequencing, namely c.182_183dupGT (p.Q62Vfs^*^48), c.2129dupC (p.A711Cfs^*^11), c.3469G>T (p.G1157^*^), c.5150_5151delAA (p.K1717Rfs^*^16) and c.1131+2_1131+5delTAAG, c.3010A>T (p.K1004^*^), c.853G>C (p.G285R). All of them were found to strongly affect the protein structure. In fact, these variants were predicted to disrupt the structure and conformation of SMCHD1 and, in most cases, alter splicing or create PTC and truncated protein products. The resulting protein has been predicted to cause the loss of *GHKL-ATPase* and *SMC hinge* domains, which allow SMCHD1 to maintain a repressive chromatin structure in muscle cells, in normal conditions [15]. These results are in line with the FSHD etiopathogenetic mechanism, which supports a toxic expression of *DUX4* as a consequence of LOF mutations in *SMCHD1* [2,17]. However, functional assays are necessary to validate the real effect of the identified variants on the protein structure and function. Interestingly, gain-of-function mutations localized in *GHKL-ATPase* domain of SMCHD1 have been shown to cause severe malformations of the human nose, olfactory tract and eyes (collectively known as Bosma arhinia microphthalmia syndrome; BAMS), whereas LOF or dominant-negative pathogenic *SMCHD1* mutations have been found throughout the sequence of the gene [22,23]. Why mutations of *SMCHD1* lead to the development of FSHD rather than BAMS is still a matter of investigation [22,23]. However, these data emphasize the importance of considering the genetic background of patients to clarify the clinical variability of such disorders. The present study showed that the analysis of the *D4Z4* fragment and *SMCHD1* sequence were crucial to confirm the clinical phenotype and accomplish a reliable genotype–phenotype correlation. Our data are consistent with Sacconi et al. [24] who suggested that a borderline *D4Z4* fragment may be considered as a risk factor or a phenotype modifier of FSHD in patients carrying *SMCHD1* causative mutations [24]. On the other hand, patients with borderline *D4Z4* fragment who were negative to *SMCHD1* analysis could not receive a clear molecular diagnosis, although they appeared phenotypically affected. This data highlights the fact that probably one or more unknown genes contribute to determining the permissive background for FSHD. On this subject, a recent study identified a potentially damaging mutation in the *DNA Methyltransferase 3 Beta* (*DNMT3B*, 20q11.21, #602900) gene, which is a *D4Z4*-chromatin modifier and, therefore, it represents a good candidate gene for FSHD [2,18,25]. In this context, we developed an NGS panel, including a set of candidate genes involved in the epigenetic regulation of the *D4Z4* region and genes targeted by *DUX4* (data in progress). Moreover, the analysis of the *SMCHD1* sequence revealed the presence of variants in the 3′UTR of the gene, which may affect the binding of specific miRNAs or their interaction with target mRNAs. In this perspective, the rs149259359 (G/C) appeared to be the most interesting among the identified 3′UTR variants. In fact, the variant allele (C) of the SNP was predicted to disrupt the binding sites of different miRNAs, including MIR95, which is known to be overexpressed during myogenic differentiation [26]. A disruption of its binding site may thereby affect the expression of MIR95 and its modulatory effect in myogenic cells, suggesting a potential role as a disease modifier in FSHD [26]. Given the fact that the variant has been identified in the patient carrying c.5150_5151delAA, we performed segregation analysis on his family members. Interestingly, rs149259359 was detected in both the affected mother and maternal uncle, supporting its potential implication in FSHD etiopathogenesis or severity. These findings support the role of epigenetics as a hallmark and/or phenotype modifier of disease [23].

Altogether, the present study highlights how NGS platforms can be helpful to disclose *SMCHD1* as well as other candidate genes involved in FSHD pathogenesis. However, NGS still needs to be always combined with labor-intensive, outdated genetic methodologies (such as southern blotting) to better characterize the complex etiopathogenetic background of FSHD [2, 20]. On this subject, the recent development of alternative molecular approaches, including molecular combing and optical mapping platforms, proved to be the most feasible alternatives for FSHD molecular diagnosis and investigation [2]. Moreover, a deeper characterization of the leading mechanisms underlying the disease can be critical for undertaking the most suitable molecular assays and allowing an accurate genotype–phenotype correlation [27–28]. In this perspective, the integration of molecular findings and clinical data is essential to develop precision medicine protocols for FSHD patients.

## Methods

### Description of patients’ cohort

The study involved 69 Italian individuals with a clinical diagnosis of FSHD enrolled at different specialized centers. Recruited patients had an average age of 50 years and a 49:51 male/female ratio. The clinical evaluation of patients was performed by specialized physicians following the dedicated guidelines [29–30]. All participants provided signed informed consent for research and publication at the time of recruitment. The study was approved by the ethics committee of Santa Lucia Foundation and complied with Declaration of Helsinki.

### DNA extraction and *D4Z4* analysis

The DNA was initially extracted from lymphocytes according to standard procedures. Successively, the extracted DNA was digested on agarose plugs by restriction enzymes (*Eco*RI, *Eco*RI/BlnI and XapI) and, subsequently, separated by pulsed-field gel electrophoresis (PFGE) as previously described [20]. The *D4Z4* size was evaluated by southern blotting and hybridization with p13E-11 probe according to standard procedures. Linear gel electrophoresis (LGE) was utilized to confirm the results. In addition, 4qA and 4qB alleles were subjected to enzymatic digestion (with *Hin*dIII and *Eco*RI), PFGE and southern blot hybridization with radiolabeled 4qB and 4qA probes, according to standard procedures [20].

### *SMCHD1* sequence analysis

*SMCHD1* gene was extensively investigated by next-generation sequencing (NGS) and direct sequencing, searching for putative variants located within the intronic, exonic and 3′UTR regions. To this purpose, the DNA was re-extracted from 400 μl of peripheral blood using MagPurix Blood DNA Extraction Kit and MagPurix Automatic Extraction System (Resnova) according to the manufacturer’s instructions. The *SMCHD1* gene was sequenced using Ion Torrent S5 and Ion AmpliSeq Customized Panel, designed by Ion AmpliSeq Designer (Thermo Fisher Scientific). The panel was expected to screen approximately 99.72% of target sequences, considering a minimum base pair coverage of 20×. The construction of the library was performed by Ion AmpliSeq™ Library Kits Plus and utilizing approximately 10 ng/μl of starting DNA for multiplex PCR reactions. Two purification steps (using AMPure XP, Beckman Coulter) were performed to remove unwanted contaminants, followed by a final PCR according to the manufacturer’s instruction. The quality of library was evaluated by Qubit R 2.0 Fluorometer (Thermo Fisher Scientific). The enrichment procedures were performed by Ion Chef System (Thermo Fisher Scientific). Ion 510™ and Ion 520™ and Ion 530™ Kit-Chef (Thermo Fisher Scientific) were utilized for template amplification, enrichment and sequencing. Samples were run on Ion 520™ Chip (850 flows required) and Ion Torrent S5 (Thermo Fisher Scientific). The sequencing run was considered of good quality if the average coverage for each sample was approximately 80–95% and the percentage of polyclonal fragments was less than 33%. NGS revealed a number between 10 and 20 variants for each sample, which were subsequently analyzed by Ion Reporter 5.6 (Thermo Fisher Scientific), Integrative Genomics Viewer (IGV). hg19 (GRCh37) was taken as reference genome building and NM_015295.2 as reference sequence for *SMCHD1*. The putative variants and *SMCHD1* sequence regions uncovered by NGS were analyzed by direct sequencing. To this purpose, 100 ng/μl of genomic DNA was amplified using the AmpliTaq Gold DNA Polymerase (Applied Biosystems) and PCR reagents in a total volume of 25 μl, following the manufacturer’s instructions. The amplified samples were sequenced using BigDye Terminator v3.1 Cycle Sequencing Kit (Thermo Fisher Scientific) and run on ABI3130xl (Applied Biosystems). Electropherograms were finally analyzed with Sequencing Analysis Software v.6 (Applied Biosystems).

### Interpretation of variants

The identified variants were firstly investigated by looking at frequencies and data reported on publicly available database (1000 Genome Browser v.3.7.6/25/07/2019, ExAC v.0.3.1/17/03/2016, Clinvar 10/07/2019, HGMD v.2019.1/06/2019, GnomAD v.2.1.1/6/03/2019). UniProt annotation database [31] was used to obtain the amino acid sequence and the protein domains of wild-type SMCHD1. The functional effect of the detected variants was evaluated by bioinformatic predictive tools, including MutationTaster, Varsome, SMART, HSF, Phyre2, VarSite and Missense3D [32–39]. In particular, MutationTaster evaluates the potential pathogenic effect of DNA sequence alterations by predicting the functional consequences of amino acid substitutions, intronic and synonymous alterations, short insertions and/or deletions (indels) and variants spanning intron–exon borders affecting splicing activity [32]. Varsome is a powerful annotation tool and search engine for human genomic variants, allowing the classification of variants according to ACMG criteria [33]. SMART, VarSite, Missense3D and Phyre2 enable the prediction of the effect of the variants on the protein structure [34–38]. In particular, SMART performs the analysis of the architecture of protein domains whereas Phyre2, VarSite and Missense3D are able to analyze the effect of amino acid changes on the protein structure, providing a 3D model of the predicted results. HSF predicts the effects of variants on the splicing mechanisms [39]. PolymiRTs Database 3.0 was used to analyze the variants detected within the 3′UTR of *SMCHD1*. It allows the evaluation of the functional impact of genetic variants located in microRNA (miRNA) seed regions and miRNA target sites, predicting the effect on the miRNA–mRNA binding [40].

Taking into account frequency and predictive results, the variants of *SMCHD1* have been classified according to the ACMG Standards and Guidelines, which facilitate the clinical interpretation of variants, by discriminating among benign, likely benign, uncertain significance, likely pathogenic and pathogenic variants [41].

## Funding

Italian Ministry of Health (5X1000-2016 and 5x2017 MINSAL.3).

## Supplementary Material

Supplementary_tables_ddz239
