# Supplementary material for: The variability of SMCHD1 gene in FSHD patients: evidence of new mutations
Source: Hum Mol Genet. 2019 Oct 10;28(23):3912–20. doi: 10.1093/hmg/ddz239 (PMC6969370; doi:10.1093/hmg/ddz239)
Supplement: Supplementary_tables_ddz239 [file supplementary_tables_ddz239.docx]

**Suppl. Table 1.** List of the 14 exonic variants detected by analysis of the *SMCHD1* sequence.

NA: Not Available. *Calculated on 69 patients, ^#^Referred to 1000Genomes allele frequencies

| **SNP** | **Coding** | **Protein** | **Genomic location** | **Exon** | **MAF FSHD*** | **MAF EUR^#^** | **ACMG** |
| --- | --- | --- | --- | --- | --- | --- | --- |
| rs2430853 | c.174G>C | p.(=) | chr18:2656249 | 1 | G: 0.377 | G: 0.450 | Benign |
| rs635132 | c.1851A>G | p.(=) | chr18:2705700 | 14 | A: 0.377 | A: 0.282 | Benign |
| rs2276092 | c.2122G>A | p.Val708Ile | chr18:2707619 | 16 | G: 0.334 | G: 0.309 | Benign |
| rs633422 | c.2637A>T | p.Lys879Asn | chr18:2724930 | 21 | T: 0.326 | T: 0.455 | Benign |
| rs12327477 | c.3528A>G | p.(=) | chr18:2740714 | 28 | G: 0.275 | G: 0.263 | Benign |
| rs2304859 | c.4137A>G | p.(=) | chr18:2750477 | 32 | G: 0.282 | G: 0.260 | Benign |
| rs483547 | c.4629C>T | p.(=) | chr18:2763697 | 37 | T: 0.326 | T: 0.460 | Benign |
| rs117771893 | c.1419A>G | p.(=) | chr18:2700613 | 11 | G: 0.007 | G: 0.005 | Likely Benign |
| NA | c.2748G>T | p.Gln916His | chr18:2726497 | 22 | T: 0.022 | NA | Uncertain Significance |
| NA | c.2748G>A | p.(=) | chr18:2726497 | 22 | A: 0.007 | NA | Uncertain Significance |
| NA | c.4310A>C | p.Lys1437Thr | chr18:2752514 | 34 | C: 0.007 | NA | Uncertain Significance |
| NA | c.5870A>G | p.Glu1957Gly | chr18:2796097 | 46 | G: 0.007 | NA | Uncertain Significance |
| rs1359616018 | c.924T>A | p.His308Gln | chr18:2694575 | 8 | A: 0.007 | A: 0.000 | Uncertain Significance |
| NA | c.3514G>T | p.Val1172Phe | chr18:2739518 | 27 | T: 0.007 | NA | Uncertain Significance |

**Suppl. Table 2.** List of the 56 intronic variants found by the screening of the *SMCHD1* sequence.

NA: Not Available. *Calculated on 69 patients, ^#^Referred to 1000Genomes allele frequencies. ^§^This frequency is referred to Non-Finnish European (NFE) Population.

| **SNP** | **Coding** | **Genomic Location** | **Type** | **MAF FSHD*** | **MAF EUR ^#^** |
| --- | --- | --- | --- | --- | --- |
| rs637651 | c.873+53G>A | chr18:2688798 | SNV | G: 0.094 | G: 0.054 |
| rs62084202 | c.1132-146A>G | chr18:2697683 | SNV | G: 0.181 | G: 0.196 |
| rs626994 | c.1342+86T>C | chr18:2698125 | SNV | T: 0.087 | T: 0.054 |
| rs481575 | c.1342+133A>G | chr18:2698172 | SNV | A: 0.065 | A: 0.024 |
| rs62084217 | c.1843-77A>G | chr18:2705615 | SNV | G: 0.196 | G: 0.193 |
| rs8090967 | c.1843-59T>A | chr18:2705633 | SNV | T: 0.493 | A: 0.421 |
| rs62084229 | c.2338+32A>G | chr18:2718265 | SNV | G: 0.210 | G: 0.197 |
| rs17627559 | c.2914-96T>C | chr18:2729177 | SNV | C: 0.203 | C: 0.197 |
| rs10638660, rs139785567,  rs397692326 | c.3277-43T>TAGAC | chr18:2738352 | INDEL | AGAC: 0.290 | AGAC: 0.263 |
| rs8094260 | c.3425+37C>T | chr18:2738580 | SNV | T: 0.268 | T: 0.263 |
| rs521992 | c.3515-76G>A | chr18:2740625 | SNV | A: 0.319 | A: 0.454 |
| rs2304862 | c.3927+151A>T | chr18:2747796 | SNV | T: 0.203 | T: 0.197 |
| rs71365197 | c.4166-110C>CAT | chr18:2751166 | INDEL | AT: 0.283 | AT: 0.263 |
| rs300293 | c.4281+84A>G | chr18:2751475 | SNV | A: 0.109 | A: 0.017 |
| rs300291 | c.4967-15G>T | chr18:2771516 | SNV | G: 0.246 | G: 0.001 |
| rs397858944,  rs35375743 | c.5176-210TG>T | chr18:2775522 | INDEL | del: 0.261 | del: 0.467 |
| rs370583815 | c.6664G>A | chr18:2803196 | SNV | A: 0.246 | A: 0.002 |
| rs4035266 | c.7156_7159delCTAT | chr18:2803687 | INDEL | CTAT: 0.116 | CTAT: 0.000 |
| rs9947882 | c.187-110A>C | chr18:2666046 | SNV | C: 0.290 | C: 0.231 |
| rs8090988 | c.1843-17T>A | chr18:2705675 | SNV | A: 0.225 | A: 0.291 |
| rs16943716 | c.2701-85C>G | chr18:2726365 | SNV | G: 0.282 | G: 0.228 |
| rs4798020 | c.3927+195A>G | chr18:2747840 | SNV | G: 0.217 | G: 0.360 |
| rs35853884 | c.5548-44TGAAA>T | chr18:2784404 | INDEL | del: 0.283 | del: 0.229 |
| rs182879069 | c.6666A>G | chr18:2803198 | SNV | G: 0.072 | G: 0.044 |
| rs373945021 | c.6685A>T | chr18:2803217 | SNV | T: 0.007 | ^§^T: 0.0003191 |
| rs113825505 | c.3514+61A>G | chr18:2739579 | SNV | G: 0.014 | G: 0.012 |
| rs2304861 | c.4007+84C>T | chr18:2750204 | SNV | T: 0.072 | T: 0.067 |
| rs200498243 | c.5367-128TGTG>T | chr18:2777676 | INDEL | del: 0.014 | del: 0.021 |
| rs621302 | c.187-72T>C | chr18:2666084 | SNV | C: 0.072 | C: 0.054 |
| rs634246 | c.262+25G>C | chr18:2666256 | SNV | C: 0.072 | C: 0.054 |
| rs531379 | c.262+48T>C | chr18:2666279 | SNV | C: 0.072 | C: 0.054 |
| rs648105 | c.424+33T>C | chr18:2667063 | SNV | C: 0.072 | C: 0.054 |
| rs73936777 | c.507+146C>T | chr18:2673508 | SNV | T: 0.007 | T: 0.000 |
| NA | c.4967-14G>T | chr18:2771517 | SNV | T: 0.007 | NA |
| rs74270564 | c.638+26_638+27delTT | chr18:2674171_  2674172 | INDEL | del: 0.036 | NA |
| rs377473058 | c.1843-29delT | chr18:2705665 | INDEL | del: 0.036 | NA |
| rs76149169,  rs67636305 | c.2913+158 delT | chr18:2728743 | INDEL | del: 0.044 | NA |
| rs766801635 | c.6662G>A | chr18:2803194 | SNV | A: 0.007 | NA |
| rs596500 | c.638+109T>C | chr18:2674254 | SNV | C: 0.007 | C: 0.024 |
| rs147527232 | c.1131+195T>G | chr18:18:2697317 | SNV | G: 0.007 | G: 0.008 |
| rs2304860 | c.4008-110A>G | chr18:2750240 | SNV | G: 0.014 | G: 0.228 |
| rs141243907 | c.754-53G>T | chr18:2688573 | SNV | T: 0.021 | T: 0.030 |
| rs146251858 | c.754-54C>T | chr18:2688573 | SNV | T: 0.021 | T: 0.030 |
| rs371106689 | c.6061C>T | chr18:2802593 | SNV | T: 0.007 | T: 0.001 |
| rs541594137 | c.507+201ATCT>A | chr18:2673563 | INDEL | del: 0.007 | del: 0.002 |
| rs10502304 | c.2913+92T>A | chr18:2728688 | SNV | A: 0.014 | A: 0.197 |
| rs762092957 | c.1843-195ATTTG>A | chr18:2705497 | INDEL | del: 0.007 | NA |
| rs760383946 | c.4008-15CT>C | chr18:2750333 | INDEL | del: 0.007 | C: 0.000 |
| rs541442202 | c.187-168T>C | chr18:2665988 | SNV | C: 0.007 | C: 0.002 |
| rs147527232 | c.1131+195T>G | chr18:2697317 | SNV | G: 0.007 | G: 0.008 |
| rs3213926 | c.5476+10A>G | chr18:2777925 | SNV | G: 0.268 | G: 0.262 |
| rs33939734 | c.8013delT | chr18:2804544 | INDEL | GT: 0.116 | GT: 0.000 |
| rs145755468 | c.1956+7C>T | chr18:2705812 | SNV | T: 0.022 | T: 0.000 |
| rs79361541 | c.507+197T>C | chr18:2673559 | SNV | C: 0.007 | C: 0.020 |
| rs1408414690 | c.4282-24C>T | chr18:2752462 | SNV | T: 0.014 | T: 0.000 |
| NA | c.4282-22T>C | chr18:2752464 | SNV | C: 0.022 | NA |
